# Calculated inflammatory markers derived from complete blood count results, along with routine laboratory and clinical data, predict treatment failure of acute peritonitis in chronic peritoneal dialysis patients

**DOI:** 10.1080/0886022X.2023.2179856

**Published:** 2023-03-13

**Authors:** Dan Zhou, Huibin Yang, Li Zeng, Wei Yang, Fujia Guo, Wenting Cui, Cong Chen, Jiayao Zhao, Shuran Wu, Ning Yang, Hongli Lin, Anchun Yin, Longkai Li

**Affiliations:** a Department of Nephrology, Liaoning Translational Medicine Center of Nephrology, First Affiliated Hospital of Dalian Medical University, Dalian, China; b College of Integrative Medicine, Dalian Medical University, Dalian, China; c Graduate School, Dalian Medical University, Dalian, China; d First Affiliated Hospital, Dalian Medical University, Dalian, China

**Keywords:** Peritoneal dialysis, peritonitis, neutrophil-to-lymphocyte ratio (NLR), platelet-to-lymphocyte ratio (PLR), hemoglobin-to-lymphocyte ratio (HLR)

## Abstract

**Background & Aims:**

Complete blood count (CBC)-derived inflammatory markers are predictive biomarkers for the prognosis of many diseases. However, there was no study on patients with peritoneal dialysis-associated peritonitis (PDAP). We aimed to investigate the value of these markers in predicting treatment failure of acute peritonitis in chronic PD patients.

**Methods:**

The records of 138 peritonitis episodes were reviewed and divided into treatment success or failure groups in a single center for 10 years. CBC-derived markers and other routine data were recorded before peritonitis treatment was initiated. Univariate and multivariate regression analyses and the receiver operating characteristic (ROC) curve about the predictors of treatment outcomes were performed.

**Results:**

Neutrophil-to-lymphocyte ratio (NLR), platelet-to-lymphocyte ratio (PLR), monocyte-to-lymphocyte ratio (MLR), systemic immune-inflammation index (SII), and derived NLR were significantly higher in the failure group. Univariate logistic regression results showed that NLR and PLR were risk factors of treatment outcomes. The backward stepwise multivariate logistic regression results demonstrated that NLR [adjusted odds ratio (aOR), 1.376; 95% confidence intervals (CI), 1.105–1.713; *p =* .004], PLR (aOR, 1.010; 95%CI, 1.004–1.017; *p =* .002) were risk factors, but hemoglobin-to-lymphocyte ratio (HLR) (aOR, 0.977; 95%CI, 0.963–0.991; *p =* .001), and SII (aOR, 0.999; 95%CI, 0.998–1.000; *p =* .040) were protective factors. A combination of age, PD vintage, Gram-positive peritonitis, staphylococcus aureus, culture-negative, NLR, PLR, HLR, and SII would improve prognostic performance. The area under this ROC curve was 0.85, higher than other factors.

**Conclusions:**

NLR, PLR, HLR, and SII were associated with PDAP outcomes. Age, PD vintage, NLR, and PLR were significant risk factors in PDAP patients.

## 1. Introduction

Peritoneal dialysis-associated peritonitis (PDAP) remains a frequent but severe complication of peritoneal dialysis (PD) and a major cause of technical failure and death [1]. The peritoneal catheter removal rate was 22% overall, and the mortality was 2%–6% in PDAP patients [2]. Therefore, accurate assessment and prediction of the prognosis in an early phase are crucial for prompt decision-making to prevent serious outcomes (even death) in clinical practice. Markers and risk factors for outcomes of treatment of peritonitis must be identified, particularly those easily obtained at the outset of the episodes of peritonitis.

Complete blood count (CBC)-derived inflammatory markers have been recently reported to predict in the prognosis of many diseases. An increased value of the neutrophil-to-lymphocyte ratio (NLR) was associated with poor prognosis in alcoholic hepatitis [3] and acute pancreatitis [4]. Some studies have found relationships between elevated platelet-to-lymphocyte ratio (PLR) and increased mortality in COVID-19-associated pneumonia [5]. The monocyte-to-lymphocyte ratio (MLR) has also been proven to predict the relapse risk of multiple sclerosis patients [6]. However, few studies focused on CBC-derived inflammatory markers in PDAP patients, and only NLR was found to be a risk factor for a poor peritonitis outcome [7]. There are other markers derived from CBC, such as PLR [8], MLR [9], platelet-to-monocyte ratio (PMR) [10], hemoglobin-to-platelet ratio (HPR) [11], hemoglobin-to-lymphocyte ratio (HLR) [12], systemic immune-inflammation index (SII) [13] and derived neutrophil-to-lymphocyte ratio (dNLR) [14]. They were mostly related to the degree of inflammatory reaction and may be potential predictors in PDAP patients despite their different roles in different diseases. Therefore, better CBC-derived markers are needed to evaluate the prognosis in PDAP patients. But there was no study on the association between all CBC-derived inflammatory markers and outcomes of peritonitis. In addition, identifying risk factors predicting a poor prognosis is also crucial for the prognosis, so predictors of PDAP outcomes should be further investigated to guide interventions.

In order to explore better markers for PDAP prognosis, we collected the data on peritonitis for 10 years in our center, investigated the role of CBC-derived inflammatory markers, and identified predictors of the prognosis.

## 2. Methods

### 2.1. Study design and participants

It was a single-center, retrospective observational study of PDAP patients at the First Affiliated Hospital of Dalian Medical University, China. The data regarding all episodes of PDAP patients from 01 January 2012 to 31 December 2021 were collected by reviewing case records. The inclusion criteria included: (1) age ≥ 18 years, (2) PD vintage ≥1 month, and (3) meeting the diagnostic criteria of PDAP [15]. The exclusion criteria included: (1) without CBC results and peritoneal effluent test results; (2) episodes in the perioperative period; (3) a current history of hemodialysis (HD) or renal transplantation; (4) diagnosis of systemic inflammatory diseases (chronic autoimmune diseases, acute infection, and surgical peritonitis) within the preceding 1 month; (5) the episode of acute cardiovascular and cerebrovascular events within the preceding 1 month; (6) diagnosis of malignant tumors; (7) treatment with glucocorticoid or immunosuppressant within the preceding 1 year; (8) treatment with aspirin, clopidogrel, low molecular weight heparin (affecting CBC); and (9) those receiving antibiotics prior to the CBC tube collection. All the patients received continuous ambulatory peritoneal dialysis (CAPD) using glucose-based and lactate-buffered PD solutions through double cuff Silastic PD catheters. Our study was consistent with the ethical principles of the revised Declaration of Helsinki. Relevant information about personal identifiers was wiped off. The study was performed under a project license (NO. PJ-KY-2019-166) granted by Ethics Committee of The First Affiliated Hospital of Dalian Medical University.

### 2.2. Basic demographic, laboratory, and clinical data

Basic demographic data, including gender, age, duration of PD, etiology of end-stage renal disease (ESRD), and bacterial culture results, were obtained from the record system of our hospital. Blood for CBC and calculation of inflammatory markers was collected before empirical antibiotic therapy was initiated. The inflammatory markers were calculated, consisting of NLR, PLR, MLR, PMR, HPR, HLR, SII, and dNLR. SII was calculated as SII = neutrophil count × platelet count/lymphocyte count [16], and dNLR was calculated as dNLR = neutrophil count/(white blood cell count – neutrophil count) [17].

### 2.3. Laboratory measurements

Dialysate effluent was collected into blood culture bottles, and sterile tubes under aseptic operation for cell counts, Gram stain, microbial culture, and drug sensitivity tests when peritonitis of PD patients was suspected [15]. Meantime, blood samples were collected in anticoagulated tubes for CBC results before empirical antibiotic therapy.

### 2.4. Diagnosis and treatment of peritonitis

Peritonitis was diagnosed according to the 2022 ISPD guidelines [15] as the presence of at least two conditions below: (1) clinical features consistent with peritonitis, that is, abdominal pain and/or cloudy dialysis effluent; (2) dialysis effluent white cell count > 100/μL or > 0.1 × 10^9^/L (after a dwell time of at least 2 h), with > 50% polymorphonuclear leukocytes; (3) positive dialysis effluent culture.

According to the guidelines, initial empiric antibiotic therapy for peritonitis covered Gram-positive (a first-generation cephalosporin or vancomycin) and Gram-negative (a third-generation cephalosporin or an aminoglycoside) organisms. The medication regimen was adjusted once the culture results and drug sensitivity test became available. According to the ISPD guide, recurrent peritonitis was defined as an episode that occurred within 4 weeks of completion of therapy of a prior episode but with a different organism, and it was recorded as one episode. Relapsing peritonitis was defined as an episode that occurred within 4 weeks of completion of therapy of a prior episode with the same organism or one sterile (culture negative) episode. However, it was recorded as one episode with the prior episode.

### 2.5. Clinical Outcomes of peritonitis

Our primary clinical outcome was treatment failure, defined as unresolved peritonitis symptoms (abdominal pain and/or cloudy dialysis effluent) and dialysis effluent white cell count (>100/μL), resulting in catheter removal, switching to hemodialysis, and death (because of peritonitis). Treatment success was defined as the disappearance of clinical features (abdominal pain and/or cloudy dialysis effluent) and normal dialysis effluent white cell count (<100/μL).

### 2.6. Statistical analysis

Normality was tested by using the Shapiro-Wilk test. As for continuous variables in the study, normally distributed data were expressed as mean ± standard deviation (SD), and skewed data were presented as median with interquartile range (IQR). Categorical variables were statistically described as numbers (n) and percentages (%). The statistical differences between treatment success and failure groups were conducted by applying Student’s *t*-test for continuous normally distributed data, Wilcoxon-Mann-Whitney test for continuous skewed variables, and Pearson’s Chi-square test or Fisher’s exact test for categorical variables.

The prediction ability of all relevant variables was determined using the area under the receiver operating characteristics curve (AUROC) analysis. The optimal cutoff values, sensitivity, and specificity, were determined by maximizing the Youden’s index (sensitivity plus specificity minus 1). The univariate logistic regression model was performed to assess the relevant markers and determine the independent markers of treatment failure. Odds ratios (ORs) and their 95% confidence intervals (CI) were calculated to determine the relationship between related factors and treatment outcomes.

The variables with a *p* value of <.2 in the univariate logistic regression analysis were selected for the multivariate logistic regression model to explore the independent protective and risk factors of treatment failure. In the multivariate logistic regression model, the backward stepwise regression procedure played a vital role in isolating the predictors. The difference between the two groups was considered statistically significant at *p* < .05. All analyses were performed using Stata Edition 15.1 (Stata Corp, College Station, TX, USA).

## 3. Results

### 3.1. Patient characteristics and episodes

**Figure 1.**
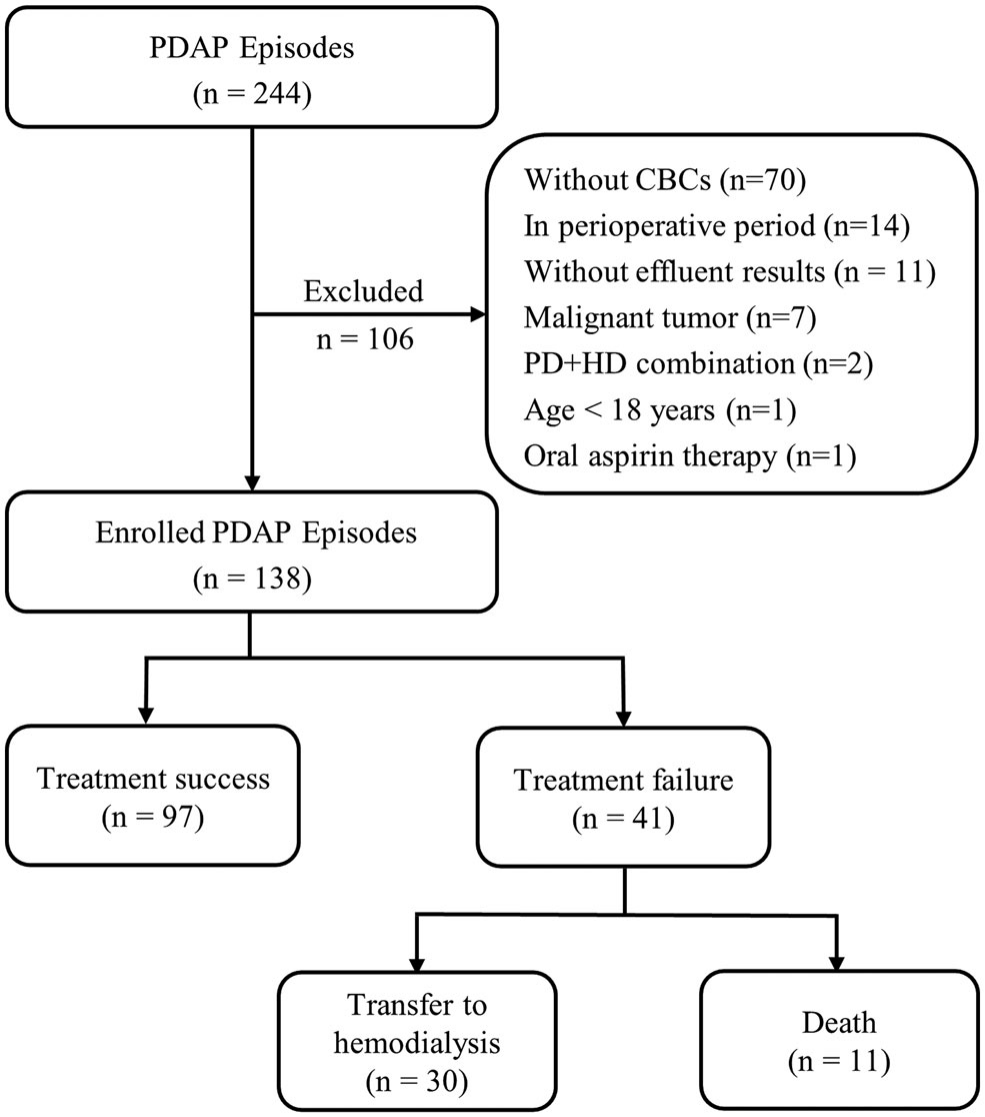
Flowchart of patient selection.

During the 10-year study period, 244 episodes of PDAP in 171 PD patients were involved in the initial cohort in the present study (Figure 1). One hundred and six episodes were excluded for the following reasons: 70 episodes were without CBC results, 14 were in the perioperative period, 11 were without effluent test results, 7 were malignant tumor patients, 2 were patients with combination PD and HD, 1 was under 18 years, and 1 was in oral aspirin therapy. One hundred thirty-eight episodes (in 116 PD patients) were eventually eligible and divided into treatment success and failure groups. Ninety-seven (70.29%) episodes (in 77 PD patients) were identified as treatment success; 41 (29.71%) episodes (in 41 PD patients) were identified as treatment failure, including 30 (21.73%) transfers to hemodialysis and 11 (7.97%) deaths (Figure 1).

Baseline characteristics are presented in Table 1. The median age was 58.44 (IQR, 42.39–69.11) years, and 57 patients (58.76%) were males in the success group. The median age was 57.71 (IQR, 49.24–71.09) years, and 24 patients (58.54%) were males in the failure group. No significant differences were observed in age and gender between the two groups. However, there was a significant difference between the success and failure groups in the PD vintage (23.10 months, IQR, 8.80–46.47 *vs.* 30.57 months, IQR, 14.30–56.57, *p* = .016). Regarding the etiology of ESRD, we analyzed diabetic nephropathy, chronic glomerulonephritis, benign arteriolar nephrosclerosis, and other diseases. No significant differences were found between the two groups. According to culture results, there were 45 (32.61%) episodes with Gram-positive organisms, 31 (22.46%) episodes with Gram-negative organisms, 7 (5.07%) episodes with fungus, and 55 (39.86%) episodes with negative cultures. There were no significant differences between the two groups except for the culture-negative peritonitis (45, 46.39% *vs.* 10, 24.39%, *p* = .022). All the episodes with different strains have also been analyzed, including Staphylococcus epidermidis, Staphylococcus aureus, Staphylococcus haemolyticus, Staphylococcus hominis, Staphylococcus auricularis, Escherichia coli, and Pseudomonas aeruginosa. No significant statistical difference was found between the success and failure groups except for Staphylococcus aureus (4, 11.76% *vs.* 5, 45.45%, *p* = .028).

**Table 1.** Characteristics of all episodes according to the treatment outcomes.

Variables	All episodes	Treatment success	Treatment failure	*p* Value
Baseline characteristics				
Number of Episodes, *n* (%)	138 (100%)	97 (70.29%)	41 (29.71%)	－
Males, *n* (%)	81 (58.70%)	57 (58.76%)	24 (58.54%)	.980
Age (years)	58.07 (44.43–70.45)	58.44 (42.39–69.11)	57.71 (49.24–71.09)	.232
Duration on PD (months)	26.42 (11.40–50.07)	23.10 (8.80–46.47)	30.57 (14.30–56.57)	***.016***
Etiology of ESRD				
Diabetic nephropathy	45 (32.61%)	31 (31.96%)	14 (34.15%)	.802
Chronic glomerulonephritis	61 (44.20%)	47 (48.45%)	14 (34.15%)	.122
Benign arteriolar nephrosclerosis	19 (13.77%)	13 (13.40%)	6 (14.63%)	.848
Others	13 (9.42%)	6 (6.19%)	7 (17.07%)	.045
Culture outcome				
Gram-positive peritonitis, *n* (%)	45 (32.61%)	34 (35.05%)	11 (26.83%)	.428
Staphylococcus epidermidis	10 (22.22%)	9 (26.47%)	1 (9.09%)	.157
Staphylococcus aureus	9 (20.00%)	4 (11.76%)	5 (45.45%)	***.028***
Staphylococcus haemolyticus	4 (8.89%)	3 (8.82%)	1 (9.09%)	.834
Staphylococcus hominis	3 (6.67%)	3 (8.82%)	0 (0)	.255
Staphylococcus auricularis	3 (6.67%)	2 (5.88%)	1 (9.09%)	.890
Others	16 (35.56%)	13 (38.24%)	3 (27.27%)	.308
Gram-negative peritonitis, *n* (%)	31 (22.46%)	18 (18.56%)	13 (31.71%)	.118
Escherichia coli	15 (48.39%)	10 (55.56%)	5 (38.46%)	.473
Pseudomonas aeruginosa	6 (19.35%)	1 (5.56%)	5 (38.46%)	.059
Others	10 (32.26%)	7 (38.89%)	3 (23.08%)	.452
Fungus, *n* (%)	7 (5.07%)	0 (0)	7 (17.07%)	***.000***
Culture-negative peritonitis, *n* (%)	55 (39.86%)	45 (46.39%)	10 (24.39%)	***.022***
Laboratory characteristics				
Effluent results (the first examination after diagnosis)				
White blood cells (/μL)	1187.5 (480–4410)	1185 (415–3400)	2200(860–6800)	***.009***
Proportion of neutrophils (%)	78 (43–90)	78 (40–87)	80 (66–93)	.058
Proportion of lymphocytes (%)	15 (6–41)	15 (8–41)	12 (4–30)	***.042***
CBC results				
White blood cell (10^9^/L)	7.53 (5.58–10.18)	7.38 (5.32–10.07)	8.85 (6.22–10.81)	.080
Neutrophil (10^9^/L)	5.81 (4.05–8.76)	5.61 (3.98–8.18)	6.91 (4.65–9.16)	.052
Lymphocyte (10^9^/L)	0.94 (0.64–1.27)	0.97 (0.70–1.32)	0.78 (0.57–1.05)	***.028***
Monocyte (10^9^/L)	0.50 (0.38–0.70)	0.47 (0.36–0.68)	0.52 (0.42–0.73)	.190
Hemoglobin (g/L)	92.01 ± 19.20	94.19 ± 16.89	82.00 ± 26.00	***.039***
Platelet (10^9^/L)	201.50 (151–246)	196 (151–237)	231 (168–271)	***.037***
NLR	5.78 (3.55–9.72)	5.13 (3.48–8.63)	8.31 (4.72–13.75)	***.003***
PLR	221.77 (146.92–319.40)	200.00 (140.37–273.42)	298.39 (160.42–446.00)	***.001***
MLR	0.55 (0.40–0.81)	0.51 (0.34–0.75)	0.68 (0.51–1.04)	***.001***
HLR	94.05 (69.39–145.24)	92.31 (69.39–134.48)	107.32 (73.33–166.10)	.252
PMR	396.18 (237.04–555.56)	398.36 (237.50–555.56)	394.00 (236.36–542.55)	.974
HPR	0.49 (0.35–0.66)	0.51 (0.39–0.66)	0.37 (0.27–0.63)	.066
SII (10^9^/L)	1229.80 (588.49–2096.35)	983.30 (520.64–1930.89)	1954.48 (957.80–3273.64)	***.001***
dNLR	3.37 (2.32–5.25)	3.05 (2.20–4.62)	4.63 (2.96–7.45)	***.002***

PD: peritoneal dialysis; ESRD: end stage renal disease; CBC: complete blood count; NLR: neutrophil-to-lymphocyte ratio; PLR: platelet-to-lymphocyte ratio; MLR: monocyte-to-lymphocyte ratio; PMR: platelet-to-monocyte ratio; HPR: hemoglobin-to-platelet ratio; HLR: hemoglobin-to-lymphocyte ratio; SII: systemic immune-inflammation index; dNLR: derived neutrophil-to-lymphocyte ratio. The results part was expressed as the median (interquartile range) and mean ± standard deviation. Bold and italic indicate *p* < .05.

### 3.2. Association between CBCs-derived inflammatory markers with treatment outcome

Analysis results of peritoneal effluent results and CBCs and CBCs-derived inflammatory markers in the patients were also presented in Table 1. No significant differences were observed in white blood cells, neutrophils, monocyte, HLR, PMR, and HPR between the two groups. The proportion of effluent lymphocytes, CBC lymphocytes, and hemoglobin was significantly higher in the success group than in the failure group. However, effluent WBC count, platelet, NLR, PLR, MLR, SII, dNLR were significantly higher in the failure group than in the success group. The violin charts also demonstrated the distribution of inflammation ratios in PDAP patients (Supplementary Figure 1).

### 3.3. The predictors in PDAP patients

To determine whether the variables were correlated with treatment failure, they were enrolled in the analysis to perform the odds ratios. As shown in Table 2, 5 variables were significant risk factors for treatment failure of PDAP, including Pseudomonas aeruginosa (OR 13.333; 95%CI, 1.506–118.060; *p* = .020), NLR (OR, 1.101; 95%CI, 1.036–1.171; *p =* .002), PD vintage (OR, 1.015; 95%CI, 1.001–1.029; *p* = .033), platelet (OR, 1.005; 95%CI, 1.001–1.010; *p =* .018), and PLR (OR, 1.005; 95%CI, 1.002–1.007; *p <* .001). Nevertheless, culture-negative (OR, 0.373; 95%CI, 0.165–0.844; *p =* .018) and hemoglobin (OR, 0.979; 95%CI, 0.960–0.999; *p =* .042) suggested good prognosis in PDAP patients.

**Table 2.** Univariate odds ratios of variables for predicting treatment outcomes in all episodes.

Variables	Odds ratio	95% confidence interval	*p* Value
Male gender	0.991	0.472–2.079	.980
Age (years)	1.016	0.992–1.041	.193
Peritoneal dialysis vintage (months)	1.015	1.001–1.029	.033*
Etiology of ESRD			
Diabetic nephropathy	1.104	0.509–2.393	.802
Chronic glomerulonephritis	0.552	0.258–1.178	.124
Benign arteriolar nephrosclerosis	1.108	0.390–3.148	.848
Others	3.123	0.979–9.955	.054
Culture results			
Gram-positive peritonitis	0.679	0.303–1.523	.094
Staphylococcus epidermidis	0.244	0.030–1.995	.188
Staphylococcus aureus	3.229	0.821–12.707	.094
Staphylococcus haemolyticus;	0.783	0.079–7.760	.835
Staphylococcus hominis	–	–	***—***
Staphylococcus auricularis	1.188	0.105–13.471	.890
Others	0.510	0.137–1.895	.315
Gram-negative peritonitis	2.038	0.885–4.690	.065
Bacterium coli	1.208	0.386–3.784	.745
Pseudomonas aeruginosa	13.333	1.506–118.060	.020*
Others	1.015	0.249–4.135	.983
Negative	0.373	0.165–0.844	.018*
Fungus	–	–	***—***
Effluent results (the first examination after diagnosis)			
White blood cells (/μL)	1.0001	1.0000–1.0002	.010*
Proportion of neutrophils	2.920	0.705–12.094	.139
Proportion of lymphocytes	0.291	0.056–1.514	.142
CBC results			
White blood cell (10^9^/L)	1.056	0.983–1.135	.138
Neutrophil (10^9^/L)	1.017	0.970–1.067	.486
Lymphocyte (10^9^/L)	0.923	0.780–1.092	.350
Monocyte (10^9^/L)	0.847	0.620–1.157	.295
Hemoglobin (g/L)	0.979	0.960–0.999	.042*
Platelet (10^9^/L)	1.005	1.001–1.010	.018*
NLR	1.101	1.036–1.171	.002**
PLR	1.005	1.002–1.007	.000**
MLR	1.818	0.933–3.540	.079
HLR	1.005	0.999–1.010	.072
PMR	0.999	0.998–1.001	.802
HPR	0.605	0.172–2.126	.434
SII	1.000	1.000–1.001	.001**
dNLR	1.099	0.985–1.225	.091

CI: confidence interval; ESRD: end stage renal disease; CBC: complete blood count; NLR: neutrophil-to-lymphocyte ratio; PLR: platelet-to-lymphocyte ratio; MLR: monocyte-to-lymphocyte ratio; PMR: platelet-to-monocyte ratio; HPR: hemoglobin-to-platelet ratio; HLR: hemoglobin-to-lymphocyte ratio; SII: systemic immune-inflammation index; dNLR: derived neutrophil-to-lymphocyte ratio. Bold and italic indicate *p* < .05, significant differences are marked by * (*p* < .05) or ** (*p* < .01).

The backward stepwise multivariate logistic regression model was further performed to investigate the risk factors of treatment outcomes (Table 3), and the results demonstrated that Staphylococcus aureus [Standard *β* value, 3.073; adjusted OR (aOR), 21.614; 95%CI, 2.829–165.144; *p =* .003) was a highly significant risk factor for treatment failure, followed by NLR (Standard *β* value, 0.319; aOR, 1.376; 95%CI, 1.105–1.713; *p =* .004), age (Standard *β* value, 0.042; aOR, 1.043; 95%CI, 1.007–1.079; *p =* .018), PD vintage (Standard *β* value, 0.021; aOR, 1.021; 95%CI, 1.003–1.040; *p =* .025) and PLR (Standard *β* value, 0.010; aOR, 1.010; 95%CI, 1.004–1.017; *p =* .002). Meanwhile, Gram-positive peritonitis (Standard *β* value, −2.606; aOR, 0.074; 95%CI, 0.019–0.290; *p <* .001) was a significant protective factor for the prognosis of PDAP, followed by culture-negative peritonitis (Standard *β* value,-1.795; aOR, 0.166; 95%CI, 0.054–0.508; *p =* .002), HLR (Standard *β* value, −0.023; aOR, 0.977; 95%CI, 0.963–0.991; *p =* .001), and SII (Standard *β* value, −0.001; aOR, 0.999; 95%CI, 0.998–1.000; *p =* .040).

**Table 3.** Predictors of treatment outcomes by stepwise multivariate logistic regression analysis in all episodes.

Variables	Standard *β* value	Odds Ratio	95% CI	*p v*alue
Age	0.042	1.043	1.007–1.079	***.018*** *
PD vintage	0.021	1.021	1.003–1.040	***.025*** *
Gram-positive peritonitis (Culture results)	–2.606	0.074	0.019–0.290	***.000*** **
Staphylococcus aureus	3.073	21.614	2.829–165.144	***.003*** **
Negative (Culture results)	–1.795	0.166	0.054–0.508	***.002*** **
NLR	0.319	1.376	1.105–1.713	***.004*** **
PLR	0.010	1.010	1.004–1.017	***.002*** **
HLR	–0.023	0.977	0.963–0.991	***.001*** **
SII	–0.001	0.999	0.998–1.000	***.040*** *

PD: peritoneal dialysis; CI: confidence interval; NLR: neutrophil-to-lymphocyte ratio; PLR: platelet-to-lymphocyte ratio; HLR: hemoglobin-to-lymphocyte ratio; SII: systemic immune-inflammation index. Bold and italic indicate *p* < .05, significant differences are marked by * (*p* < .05) or ** (*p* < .01).

**Figure 2.**
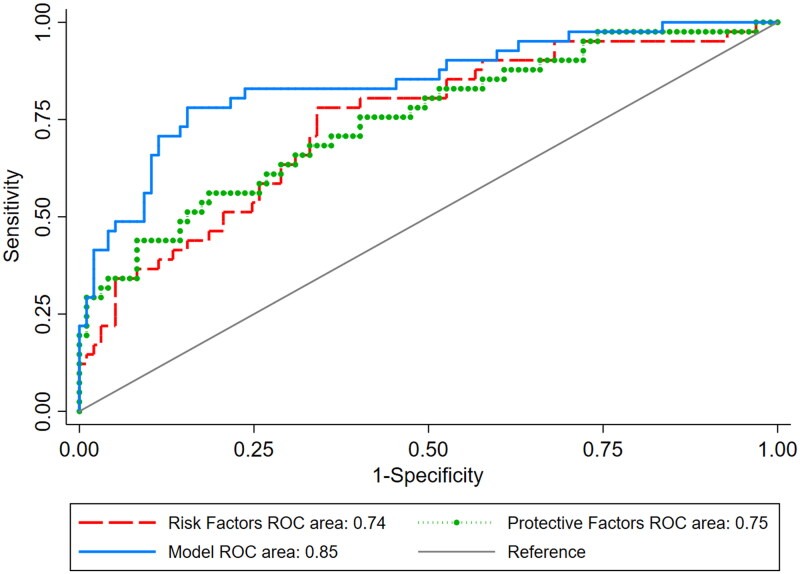
Area under the curve (AUC) of the receiver operating characteristics (ROCs) summarize the diagnostic capability of all parameters (age, PD vintage, Gram-positive peritonitis, Staphylococcus aureus, culture-negative, NLR, PLR, HLR, and SII), risk factors (Staphylococcus aureus, NLR, age, PD vintage, PLR), and protective factors (Gram-positive peritonitis, culture-negative peritonitis, HLR, SII).

In order to determine whether the combination of the 9 variables (age, PD vintage, Gram-positive peritonitis, Staphylococcus aureus, culture-negative, NLR, PLR, HLR, and SII) would improve their prognostic performance, ROC curves were plotted, and presented in Figure 2. Meantime, two ROC curves of 5 risk factors (Staphylococcus aureus, NLR, age, PD vintage, PLR) and 4 protective factors (Gram-positive peritonitis, culture-negative peritonitis, HLR, SII) were also plotted, and presented in Figure 2 according to the multivariate logistic regression analysis about risk factors and protective factors (Supplementary Table 1 and 2). The area under this ROC curve (9 factors) was 0.85, higher than any of the independent indicators (Supplementary Table 3), 5 risk factors (0.74), and 4 protective factors (0.75) (Figure 2), suggesting that the combination had a better predictor of prognosis for PADP patients.

## 4. Discussion

This study demonstrated that NLR, PLR, HLR, and SII were associated with PDAP outcomes among all CBC-derived inflammatory markers. In addition, our study showed that a combination of age, PD vintage, Gram-positive peritonitis, Staphylococcus aureus, negative culture, NLR, PLR, HLR, and SII might improve their prognostic performance in PDAP patients.

Inflammation states are expected in PDAP patients, and the degree of inflammation is closely related to unfavorable clinical outcomes [18]. Currently, clinicians pay more attention to the role of CBC-derived inflammatory markers (NLR, PLR, MLR, PMR, HPR, HLR, SII, and dNLR) in reflecting the dynamic relationship between many hematological parameters during various pathological states. Previous study results showed that these inflammatory markers were predictors in many inflammatory diseases [7–14]. However, no studies were carried out about the role of all inflammatory markers in PDAP patients except NLR. NLR was observed as a risk factor for treatment failure in PDAP patients, and those with NLR > 6.53 had a 3.41-fold increased risk of treatment failure compared to those with NLR < 3.75 [7].

PLR has been studied on overall survival rates, and the 1-year and 3-year overall survival rates for PD patients with PLR > 257.50 were 9.7% and 14.5% lower than those with PLR < 108.33 [19]. Another study result showed that the mortality rate was higher in patients with PLR ≥ 149.28 than in patients with PLR < 149.28 in PD patients [20]. Therefore, PLR was confirmed to be a risk factor regarding the clinical outcomes of PD patients, which is the same for PDAP patients in our study. Increased NLR and PLR were both significantly associated with unfavorable clinical outcomes in PDAP patients in our study. The reasons why NLR and PLR were risk factors for poor clinical prognosis may be the different responses of neutrophils and lymphocytes. Increased neutrophil counts are the main monitoring results in traditional inflammatory infection, which have been widely used in clinics [21,22]. However, previous studies have demonstrated that endocrine stress caused by inflammation will lead to the redistribution of lymphocytes from peripheral blood to lymphoid tissues, resulting in the decrease of peripheral blood lymphocytes [23–25]. Therefore, as an indicator of neutrophil-lymphocyte combination, NLR can better reflect the changes in systemic immunity and endocrine stress. Similarly, PLR represents the ratio of platelet counts to lymphocyte counts, and platelets are also a common inflammatory marker [26,27], so PLR can also reflect the changes in systemic inflammation [8]. This is consistent with our research results, suggesting NLR and PLR as risk factors in PDAP patients.

HLR and SII have not been studied in PD patients. In previous studies, they were considered risk factors in cancer research [12]. However, our study results show that they may be protective factors for prognosis in PDAP patients, suggesting their different roles in different diseases. In tumor patients, there is a special cancer-related inflammation, which manifests as a stronger neutrophil-based pro-tumor inflammatory response than a lymphocyte-based anti-tumor inflammatory response in the peripheral blood [28]. However, considering that the tumor is a chronic disease and peritonitis is an acute infection, there is no complicated tumor reaction in acute inflammation in patients with peritonitis. Meanwhile, PD patients have symptoms of renal failure, and this may affect the change of hemoglobin amount in the inflammatory reaction of peritonitis. Therefore, HLR and SII may have different roles due to different diseases.

For the protective predictors, previous studies have reported that culture-negative peritonitis may have better outcomes than that culture-positive peritonitis. The study by Magid Fahim showed that PDAP patients with negative culture had lower catheter removal (12% *vs.* 23%), permanent hemodialysis transfer (10% *vs.* 19%), and death (1% *vs.* 2.5%) compared with those with positive culture [29]. In our study, the prognosis of PD patients with negative culture peritonitis was also better than those with positive culture peritonitis. Since the negative culture rate is 39.86% (higher than the 15% suggested by ISPD) in the study, the better prognosis in patients with negative culture may be due to the use of antibiotics before peritoneal sample collection. Because many patients live far away from our center, they were given immediate antibiotics treatment from local doctors once peritonitis was considered, resulting in a high rate of negative culture results but with a good prognosis.

In addition, the combination of markers for prognosis prediction has become increasingly attractive. An Italian study demonstrated that the combination of leukocytes and peritoneal neutrophil gelatinase-associated lipocalin had a higher value in predicting PDAP [30]. A study from China showed a new risk score system (a combination of high-density lipoprotein, fibrinogen, PD duration, intestinal obstruction, diabetes mellitus, fungal peritonitis, and hemodialysis history) based on predictors they had found in PDAP patients [31]. In our study, the combination of age, PD vintage, Gram-positive peritonitis, staphylococcus aureus, negative culture, NLR, PLR, HLR, and SII was a superior independent prognostic predictor, with a prediction accuracy of 0.85, which showed better performance than other independent indicators and the 5 risk factors and the 4 protective factors. This combination may be more straightforward and effective than the previous combination.

There are also some limitations to our study. First, it was a single-center retrospective study without healthy subjects, and the sample size may have needed to be increased, although all the data were collected in the past 10 years. Second, further analysis of the CBC-derived inflammatory markers was not performed in different bacteria peritonitis episode groups because of limited episodes. Third, only the pretreatment CBC-derived inflammatory markers were assessed, but there was no continuous and dynamic monitoring during the whole treatment of peritonitis. Thus, prospective clinical trials are required to verify our current findings in the future. Notwithstanding these limitations, our study provides the first comprehensive evidence in PDAP patients on the prognostic value of CBC-derived inflammatory markers.

In conclusion, our results demonstrate that NLR, PLR, HLR, and SII were associated with PDAP outcomes among all CBC-derived inflammatory markers. Combining age, PD vintage, Gram-positive peritonitis, staphylococcus aureus, culture-negative, NLR, PLR, HLR, and SII may improve their prognostic performance in PDAP patients. However, multiple-center, prospective and large-sample studies are needed to confirm the findings.

## Supplementary Material

Supplemental Material

Supplemental Material

## References

[1] Szeto CC, Li PK. Peritoneal dialysis-associated peritonitis. Clin J Am Soc Nephrol. 2019;14(7):1100–1105. 31068338 10.2215/CJN.14631218 PMC6625612

[2] Mehrotra R, Devuyst O, Davies SJ, et al. The current state of peritoneal dialysis. J Am Soc Nephrol. 2016;27(11):3238–3252. 27339663 10.1681/ASN.2016010112 PMC5084899

[3] Forrest EH, Storey N, Sinha R, et al. Baseline neutrophil-to-lymphocyte ratio predicts response to corticosteroids and is associated with infection and renal dysfunction in alcoholic hepatitis. Aliment Pharmacol Ther. 2019;50(4):442–453. 31313853 10.1111/apt.15335

[4] Li Y, Zhao Y, Feng L, et al. Comparison of the prognostic values of inflammation markers in patients with acute pancreatitis: a retrospective cohort study. BMJ Open. 2017;7(3):e013206. 10.1136/bmjopen-2016-013206 PMC5372142 28348184

[5] Carpio-Orantes LD, García-Méndez S, Hernández-Hernández SN. Neutrophil-to-lymphocyte ratio, platelet-to-lymphocyte ratio and systemic immune-inflammation index in patients with COVID-19-associated pneumonia. Gac Med Mex. 2020;156(6):527–531. 33877106 10.24875/GMM.M21000480

[6] Huang WC, Lin HC, Yang YH, et al. Neutrophil-to-lymphocyte ratio and monocyte-to-lymphocyte ratio are associated with a 2-year relapse in patients with multiple sclerosis. Mult Scler Relat Disord. 2022;58:103514. 35032880 10.1016/j.msard.2022.103514

[7] He P, He LJ, Huang C, et al. Neutrophil-to-Lymphocyte ratio and treatment failure in peritoneal dialysis-associated peritonitis. Front Med. 2021;8:699502. 10.3389/fmed.2021.699502 PMC8350030 34381800

[8] Gasparyan AY, Ayvazyan L, Mukanova U, et al. The platelet-to-Lymphocyte ratio as an inflammatory marker in rheumatic diseases. Ann Lab Med. 2019;39(4):345–357. 30809980 10.3343/alm.2019.39.4.345 PMC6400713

[9] Demirbaş A, Elmas ÖF, Atasoy M, et al. Can monocyte to HDL cholesterol ratio and monocyte to lymphocyte ratio be markers for inflammation and oxidative stress in patients with vitiligo? A preliminary study. Arch Dermatol Res. 2021;313(6):491–498. 32816078 10.1007/s00403-020-02129-3

[10] Sierra-Rodero B, Cruz-Bermúdez A, Nadal E, et al. Clinical and molecular parameters associated to pneumonitis development in non-small-cell lung cancer patients receiving chemoimmunotherapy from NADIM trial. J Immunother Cancer. 2021;9(8):4. 10.1136/jitc-2021-002804 PMC8395363 34446577

[11] Albisinni S, Moussa I, Aoun F, et al. The impact of postoperative inflammatory biomarkers on oncologic outcomes of bladder cancer. Prog Urol. 2019;29(5):270–281. 30954405 10.1016/j.purol.2019.02.008

[12] Nanava N, Betaneli M, Giorgobiani G, et al. Complete blood count derived inflammatory biomarkers in patients with hematologic malignancies. Georgian Med News. 2020;2020(302):39–44. 32672687

[13] Liu X, Guan G, Cui X, et al. Systemic Immune-Inflammation index (SII) can be an early indicator for predicting the severity of acute pancreatitis: a retrospective study. Int J Gen Med. 2021;14:9483–9489. 34949937 10.2147/IJGM.S343110 PMC8689009

[14] Hong H, Fang X, Huang H, et al. The derived neutrophil-to-lymphocyte ratio is an independent prognostic factor in patients with angioimmunoblastic T-cell lymphoma. Br J Haematol. 2020;189(5):908–912. 32103494 10.1111/bjh.16447

[15] Li PK, Chow KM, Cho Y, et al. ISPD peritonitis guideline recommendations: 2022 update on prevention and treatment. Perit Dial Int. 2022;42(2):110–153. 35264029 10.1177/08968608221080586

[16] Huang H, Liu Q, Zhu L, et al. Prognostic value of preoperative systemic Immune-Inflammation index in patients with cervical cancer. Sci Rep. 2019;9(1):3284. 30824727 10.1038/s41598-019-39150-0 PMC6397230

[17] Alessi JV, Ricciuti B, Alden SL, et al. Low peripheral blood derived neutrophil-to-lymphocyte ratio (dNLR) is associated with increased tumor T-cell infiltration and favorable outcomes to first-line pembrolizumab in non-small cell lung cancer. J Immunother Cancer. 2021;9(11):003536. 10.1136/jitc-2021-003536 PMC8627393 34824161

[18] Kalantar-Zadeh K, Kopple JD. Relative contributions of nutrition and inflammation to clinical outcome in dialysis patients. Am J Kidney Dis. 2001;38(6):1343–1350. 11728973 10.1053/ajkd.2001.29250

[19] Liu S, Yang M, Zhao Q, et al. Platelet-to-Lymphocyte ratio is associated with the mortality in peritoneal dialysis patients. Iran J Kidney Dis. 2021;15(3):206–212. 33994380

[20] Xu LC, Zhou FF, Li M, et al. Predictive value of peripheral blood neutrophil-to-lymphocyte ratio and Platelet-To-Lymphocyte ratio on patient survival with peritoneal dialysis. Clin Lab. 2021;67(9):e210124. 10.7754/Clin.Lab.2021.210124 34542957

[21] Karon BS, Tolan NV, Wockenfus AM, et al. Evaluation of lactate, white blood cell count, neutrophil count, procalcitonin and immature granulocyte count as biomarkers for sepsis in emergency department patients. Clin Biochem. 2017;50(16–17):956–958. 28552399 10.1016/j.clinbiochem.2017.05.014

[22] Sun Y, Jiang L, Shao X. Predictive value of procalcitonin for diagnosis of infections in patients with chronic kidney disease: a comparison with traditional inflammatory markers C-reactive protein, white blood cell count, and neutrophil percentage. Int Urol Nephrol. 2017;49(12):2205–2216. 28956241 10.1007/s11255-017-1710-z

[23] Terpos E, Ntanasis-Stathopoulos I, Elalamy I, et al. Hematological findings and complications of COVID-19. Am J Hematol. 2020;95(7):834–847. 32282949 10.1002/ajh.25829 PMC7262337

[24] Reiske L, Schmucker S, Pfaffinger B, et al. Intravenous infusion of cortisol, adrenaline, or noradrenaline alters porcine immune cell numbers and promotes innate over adaptive immune functionality. J Immunol. 2020;204(12):3205–3216. 32393511 10.4049/jimmunol.2000269

[25] Zahorec R. Neutrophil-to-lymphocyte ratio, past, present and future perspectives. Bratisl Lek Listy. 2021;122(7):474–488. 34161115 10.4149/BLL_2021_078

[26] Bakogiannis C, Sachse M, Stamatelopoulos K, et al. Platelet-derived chemokines in inflammation and atherosclerosis. Cytokine. 2019;122:154157. 29198385 10.1016/j.cyto.2017.09.013

[27] Delaney C, Davizon-Castillo P, Allawzi A, et al. Platelet activation contributes to hypoxia-induced inflammation. Am J Physiol Lung Cell Mol Physiol. 2021;320(3):L413–l421. 33264579 10.1152/ajplung.00519.2020 PMC8294621

[28] Fang T, Wang Y, Yin X, et al. Diagnostic sensitivity of NLR and PLR in early diagnosis of gastric cancer. J Immunol Res. 2020;2020:9146042. 32211444 10.1155/2020/9146042 PMC7081040

[29] Fahim M, Hawley CM, McDonald SP, et al. Culture-negative peritonitis in peritoneal dialysis patients in Australia: predictors, treatment, and outcomes in 435 cases. Am J Kidney Dis. 2010;55(4):690–697. 20110144 10.1053/j.ajkd.2009.11.015

[30] Martino F, Scalzotto E, Giavarina D, et al. The role of NGAL in peritoneal dialysis effluent in early diagnosis of peritonitis: case-Control study in peritoneal dialysis patients. Perit Dial Int. 2015;35(5):559–565. 25395499 10.3747/pdi.2013.00300 PMC4597989

[31] Liu X, Qin A, Zhou H, et al. Novel predictors and risk score of treatment failure in peritoneal Dialysis-Related peritonitis. Front Med. 2021;8:639744. 10.3389/fmed.2021.639744 PMC8033636 33842502

