# Supplementary material for: Calculated inflammatory markers derived from complete blood count results, along with routine laboratory and clinical data, predict treatment failure of acute peritonitis in chronic peritoneal dialysis patients
Source: Ren Fail. 2023 Mar 13;45(1):2179856. doi: 10.1080/0886022X.2023.2179856 (PMC10013372; doi:10.1080/0886022X.2023.2179856)
Supplement: Supplemental Material [file IRNF_A_2179856_SM1574.pdf]

Supplementary Table 1 Risk Factors of treatment outcomes by multivariate logistic regression analysis in all episodes

**Variables**	**Odds Ratio**	**95% CI**	***p* value**
Age	1.024	0.996-1.052	0.089
PD vintage	1.013	0.997-1.028	0.116
Staphylococcus aureus	3.201	0.701-14.619	0.133
NLR	1.043	0.967-1.125	0.274
PLR	1.003	1.000-1.006	***0.029****

PD: peritoneal dialysis; CI: confidence interval; NLR: neutrophil-to-lymphocyte ratio; PLR: platelet-to-lymphocyte ratio. Bold and italic indicate $p < 0.05$, significant differences are marked by * ($p < 0.05$) or ** ($p < 0.01$).

Supplementary Table 2 Protective Factors of treatment outcomes by multivariate logistic regression analysis in all episodes

**Variables**	**Odds Ratio**	**95% CI**	***p* value**
Gram-positive peritonitis (Culture results)	0.277	0.103-0.746	***0.011****
Negative (Culture results)	0.216	0.081-0.577	***0.002*****
HLR	1.001	0.995-1.006	0.844
SII	1.002	1.000-1.003	***0.004*****

CI: confidence interval; HLR: hemoglobin-to-lymphocyte ratio; SII: systemic immune-inflammation index. Bold and italic indicate $p < 0.05$, significant differences are marked by * ($p < 0.05$) or ** ($p < 0.01$).

Supplementary Table 3 Cut-off values, sensitivity, specificity and receiver operating characteristics (ROC) curves of variables for predicting treatment outcomes of peritonitis

Variables	cut-off	sensibility	specificity	AUC (95% CI)
Age (years)	47.479	0.83	0.33	0.56 (0.46-0.67)
Duration on peritoneal dialysis (months)	49.183	0.46	0.80	0.63 (0.53-0.73)
Effluent results (the first examination after diagnosis)				
White blood cells (/μl)	4160.000	0.46	0.82	0.64 (0.54-0.74)
Proportion of neutrophils (%)	90.500	0.34	0.87	0.60 (0.49-0.71)
Proportion of lymphocytes (%)	6.500	0.80	0.41	0.61 (0.50-0.72)
CBC results				
White blood cell (10^9/L)	7.880	0.63	0.63	0.59 (0.49-0.70)
Neutrophil (10^9/L)	6.835	0.54	0.70	0.61 (0.50-0.71)
Lymphocyte (10^9/L)	0.655	0.81	0.41	0.62 (0.51-0.72)
Monocyte (10^9/L)	0.495	0.63	0.56	0.57 (0.47-0.67)
Hemoglobin (g/L)	83.500	0.77	0.59	0.65 (0.54-0.76)
Platelet (10^9/L)	218.500	0.56	0.71	0.61 (0.50-0.73)
NLR	5.785	0.71	0.59	0.66 (0.56-0.76)
PLR	250.693	0.68	0.67	0.68 (0.57-0.79)
MLR	0.614	0.66	0.68	0.68 (0.58-0.78)
HLR	130.323	0.44	0.73	0.56 (0.45-0.67)
PMR	46.396	1.00	0.08	0.50 (0.40-0.61)
HPR	0.310	0.77	0.54	0.60 (0.48-0.72)
SII	773.388	0.88	0.41	0.68 (0.57-0.78)
dNLR	3.439	0.71	0.61	0.67 (0.57-0.77)

CI: confidence interval; CBC: complete blood count; NLR: neutrophil-to-lymphocyte ratio; PLR: platelet-to-lymphocyte ratio; MLR: monocyte-to-lymphocyte ratio; PMR: platelet-to-monocyte ratio; HPR: hemoglobin-to-platelet ratio; HLR: hemoglobin-to-lymphocyte ratio; SII: systemic immune-inflammation index; dNLR: derived neutrophil-to-lymphocyte ratio. Bold and italic indicate $p < 0.05$, significant differences are marked by * ($p < 0.05$) or ** ($p < 0.01$).
