# Supplementary figures and images for: Calculated inflammatory markers derived from complete blood count results, along with routine laboratory and clinical data, predict treatment failure of acute peritonitis in chronic peritoneal dialysis patients
Source: Ren Fail. 2023 Mar 13;45(1):2179856. doi: 10.1080/0886022X.2023.2179856 (PMC10013372; doi:10.1080/0886022X.2023.2179856)

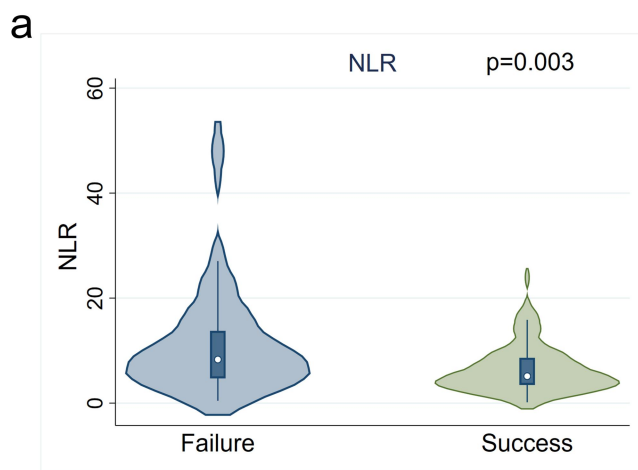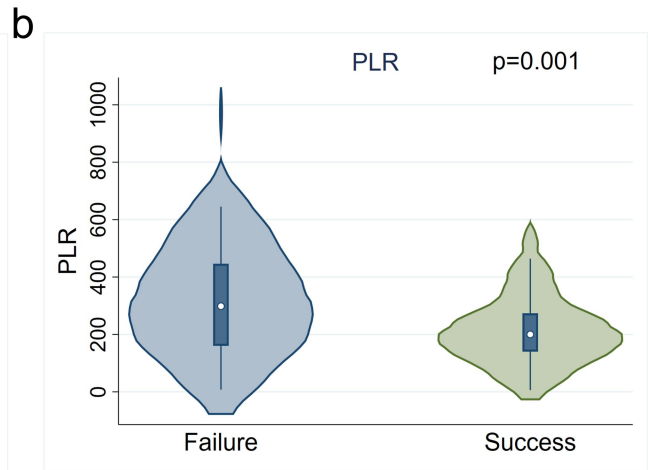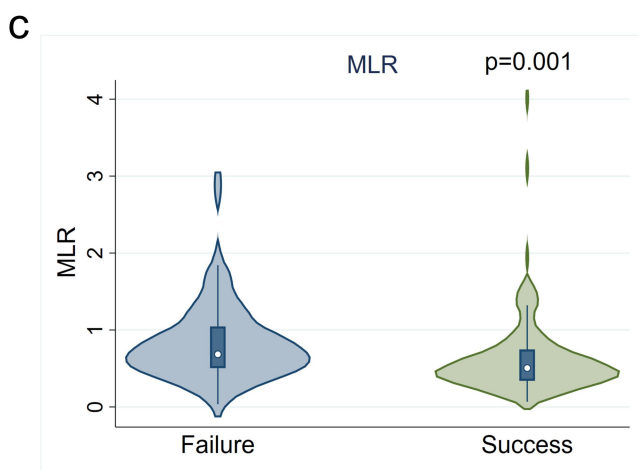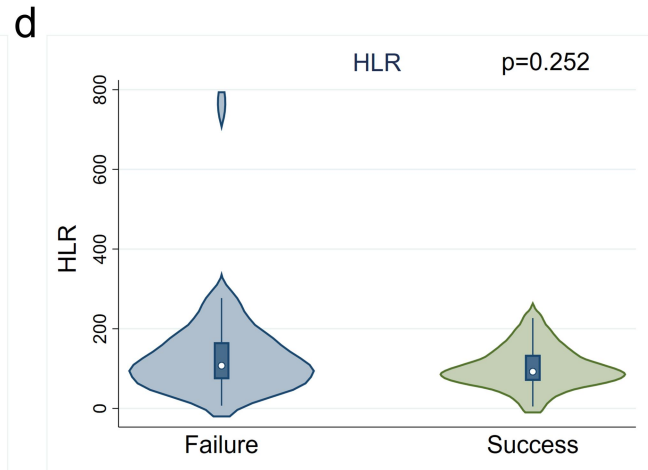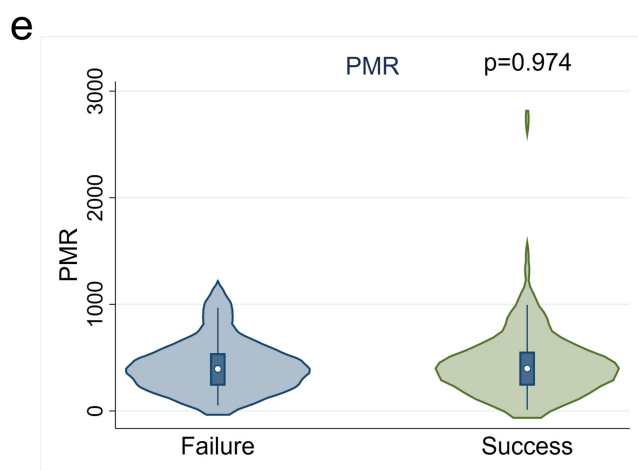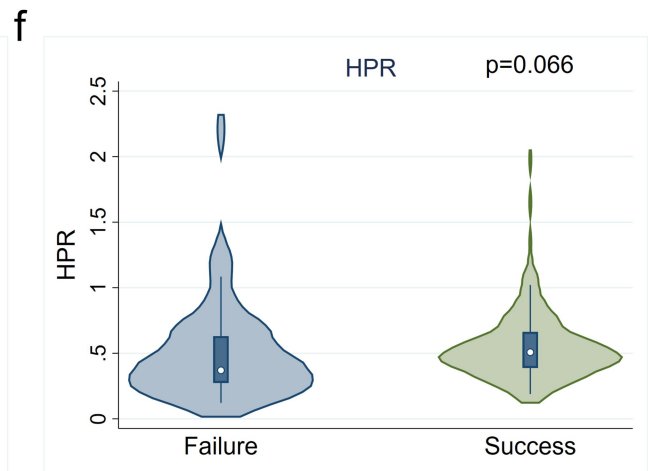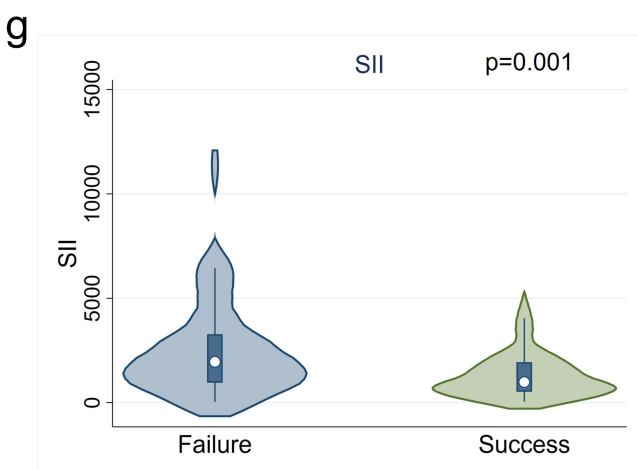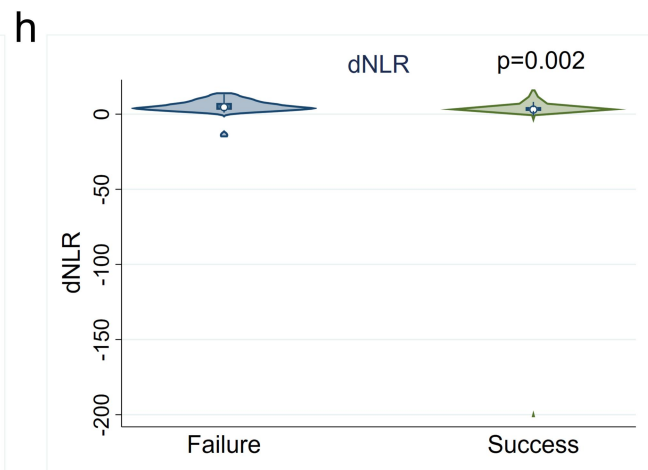

Supplement: Supplemental Material [file IRNF_A_2179856_SM1564.pdf]
